# APG101 efficiently rescues erythropoiesis in lower risk myelodysplastic syndromes with severe impairment of hematopoiesis

**DOI:** 10.18632/oncotarget.7469

**Published:** 2016-02-18

**Authors:** Anna Raimbault, Cecile Pierre-Eugene, Alexandra Rouquette, Celine Deudon, Lise Willems, Nicolas Chapuis, Stephanie Mathis, Claudia Kunz, Harald Fricke, Olivier Kosmider, Valerie Bardet, Michaela Fontenay

**Affiliations:** ^1^ Assistance Publique-Hôpitaux de Paris, Service d'Hématologie Biologique, Hôpitaux Universitaires Paris Centre, Hôpital Cochin, Paris, France; ^2^ Université Paris Descartes, Faculté de Médecine, Paris, France; ^3^ Institut National de la Santé et de la Recherche Médicale (INSERM) U1016, Paris, France; ^4^ Centre National de la Recherche Scientifique, Unité Mixte de Recherche 8104, Paris, France; ^5^ Institut Cochin, Department of Development, Reproduction and Cancer, Paris, France; ^6^ Département d'Epidémiologie et de Biostatistiques, Hôpitaux Universitaires Paris Centre, Paris, France; ^7^ Assistance Publique-Hôpitaux de Paris, Service d'Hématologie Clinique, Hôpitaux Universitaires Paris Centre, Hôpital Cochin, Paris, France; ^8^ APOGENIX, GmbH, Heidelberg, Germany

**Keywords:** myelodysplastic syndromes, erythropoiesis, CD95, CD95 ligand, anemia

## Abstract

CD95, a member of the death receptor family initiates a caspase-dependent apoptosis, when activated by its ligand CD95L, thought to negatively regulate erythrocyte production in the bone marrow. We have previously shown that CD95 is overexpressed in two thirds of patients with a lower risk myelodysplastic syndrome (MDS) and that resistance to erythropoiesis-stimulating agents (ESA) is linked to poor residual erythropoiesis. In the present study, we show that CD95 overexpression and previous transfusion are independent predictive factors of ESA resistance. To investigate an alternative therapeutic strategy of anemia in ESA-resistant patients, we have conducted a preclinical study of the effects of APG101, a fusion protein consisting of the extracellular domain of human CD95 and the Fc region of human IgG1 on MDS erythropoiesis *in vitro*. APG101 increases the number of burst-forming unit-erythroid (BFU-E) progenitors derived from CD34^+^ progenitors in liquid culture and improves overall proliferation rate of erythroid precursors by inhibiting apoptosis. APG101 rescues BFU-E growth in MDS patients presenting with attrition of erythroid progenitors at baseline, independently of CD95 or CD95L expression level. Our data show that overexpression of CD95 at diagnosis is a hallmark of ESA resistance and that severe impairment of erythropoiesis is predictive of erythroid response to APG101 *in vitro*. These data provide a rationale for further clinical investigation of APG101 in an attempt to treat anemia in lower risk MDS patients.

## INTRODUCTION

CD95 (Fas, APO-1, TNFRSF6, APT1) belongs to the death receptor family. When bound to its ligand CD95L (FasL), it initiates the assembly of a signaling complex that activates initiating caspase 8 or 10 and leads to apoptosis. The CD95 pathway is essential to eliminate auto-reactive B lymphocytes, controlling T cell homeostasis and negatively regulating erythrocyte, neutrophil and megakaryocyte production by inducing cell death in the bone marrow. [1] CD95-dependent apoptosis is thought to occur in the erythroblastic island when the erythropoietin concentration decreases locally due to the expansion of mature erythroblasts which trap the hormone. CD95L expressed on the more mature erythroblasts induces apoptosis of the immature erythroblasts expressing CD95. [2, 3] We have previously reported that CD95 is overexpressed on CD34^+^ progenitors and erythroblasts in two thirds of patients with lower risk myelodysplastic syndromes (MDS), a clonal disorder of the hematopoietic stem cell (HSC) that evolves to acute myeloid leukemia (AML) in 30% of cases. Furthermore, the expression of CD95L becomes upregulated in mature erythroblastic precursors during *in vitro* differentiation of CD34^+^ progenitors. Inhibition of CD95 activation prevents massive apoptosis of immature erythroblastic precursors that is at least partly responsible for peripheral blood anemia. [4, 5]

In early MDS, the aim of treatments is to correct cytopenias. Therefore, erythropoiesis-stimulating agents (ESA) are largely used despite these treatments are not yet approved by the FDA/EMEA in this indication. Less than 500 UI/L serum erythropoietin (EPO) level and less than 2 red blood cell (RBC) units *per* month were associated with the best response rates that hardly reached 39 to 62% in terms of transfusion independency or hematological improvement of the erythroid lineage (HI-E) according to IWG criteria. [6, 7, 8, 9] A decision model based on these two parameters has been proposed and validated by the Nordic group, [8] and has recently been improved by the addition of flow cytometry score. [10] Significantly higher response rate was observed in MDS with less than 10% blasts, low and int-1 International Prognostic Scoring System (IPSS), and red blood cell (RBC) transfusion independence in studies from the Groupe Francophone des Myélodysplasies. [9] In this cohort, a shorter interval between diagnosis and treatment could support a positive impact of ESA on overall survival. [9, 11, 12] However, limited response rate suggests that other factors may influence the response. We have previously identified severe impairment of erythropoiesis as a hallmark of patients who will fail to response to ESA. [13] Thus, to cure anemia in these patients, alternative therapeutic strategies are necessary.

In the present observational study, a preclinical research was performed to investigate the impact of the recombinant glycosylated fusion protein, APG101, consisting of the extracellular domain of human CD95 linked to the Fc domain of human IgG1 (CD95-Fc) on erythropoiesis in patients with lower risk MDS *in vitro*. APG101 effectively binds to CD95L expressed on target cells as well as to functionally active ligand in solution, and thus inhibiting CD95 activation. We show here that APG101 rescued erythropoiesis in MDS patients with impaired erythropoiesis independently of the expression level of CD95 or CD95L.

## RESULTS

### CD95, but not CD95L is overexpressed in lower risk MDS

**Figure 1:**
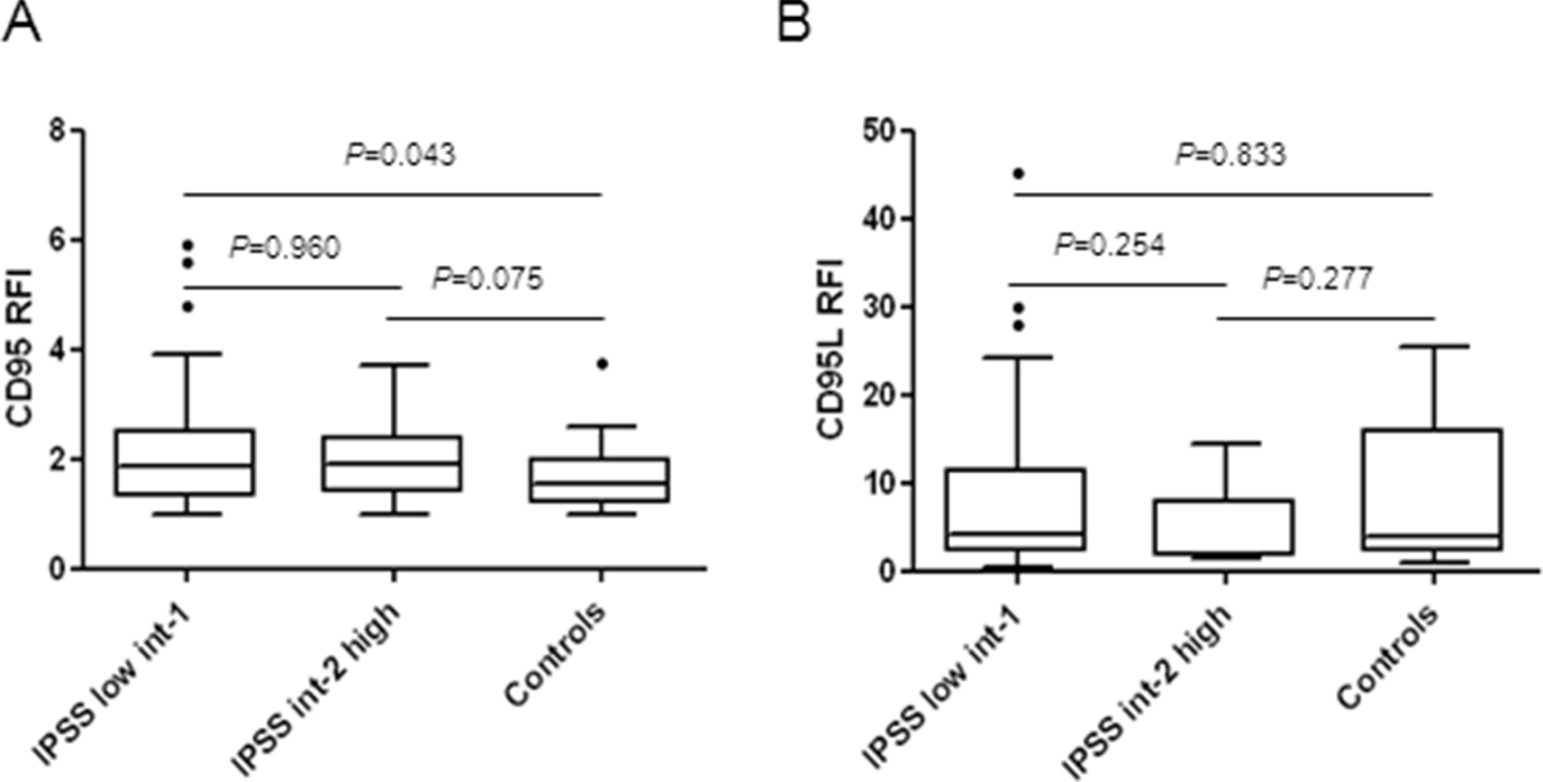
**A.** Membrane CD95 expression in MDS bone marrow. CD95 expression was quantified in the CD45^low^ cell population by flow cytometry. Results as ratios of fluorescence intensity (RFI) in 30 controls and 192 patients including 162 low/int-1 and 30 int-2/high MDS. **B.** Membrane expression of CD95 ligand (CD95L) in MDS bone marrows. CD95L expression was quantified in the CD45^low^ cell population. Results as RFI in 41 low/in-1 and 9 int-2/high MDS patients and 18 controls. Box plots with horizontal bars indicating the median and 1^st^ and 99^th^ centiles. Unpaired Student t-test or Mann-Whitney-test for *P* values.

We compared the expression level of CD95 on CD45^low^ bone marrow cells in a large cohort of 250 MDS including 162 low/int-1 IPSS and 30 int-2/high IPSS MDS before treatment (Supplementary Table S1) and 30 controls. Median CD95 RFI level was significantly higher in IPSS low/int-1 MDS compared to controls (*P* = 0.043) (Figure 1A). Accordingly, the median value of CD95 was also significantly more elevated in very high/high IPSS-R subgroup compared to controls (*P* = 0.023) and had a trend to be also higher in very low/low/intermediate IPSS-R subgroups (*P* = 0.054) (Supplementary Figure S1A).

We also quantified the expression of membrane-bound CD95L on CD45^low^ bone marrow cells in 82 MDS before treatment and 18 controls (Supplementary Table S1). We found that the median RFI of CD95L was similar between IPSS low/int-1 MDS, int-2/high MDS and controls groups (Figure 1B) and similar between the IPSS-R very low/low/intermediate, very high/high and controls groups (Supplementary Figure S1B).

### Genetic control of CD95 expression

Two single nucleotide polymorphisms −1377 (rs2234767) G>A and −670 (rs1800682) A>G located in the promoter core of the gene have been reported to control CD95 expression level. [15, 16, 17, 18] To investigate this point in MDS, we screened 129 samples from this cohort for their polymorphism at −377 and −670 loci. The frequency of −1377A allele appeared to be less represented in MDS (10.8%) compared to the current frequency in Caucasians (15%) while the −670G allele was equally represented in MDS (49.2%) compared to controls (49.0%). [15, 17, 18] We then compared CD95 expression level in each genotype. We found that the median RFI value was not significantly different between the −1377 GG *vs*-1377 GA/AA genotypes (Mann-Whitney test, *P=0.118*). By contrast, the median CD95 RFI was significantly higher in the group with −670 AA genotype compared to the group with −670 AG/GG genotypes (Mann-Whitney test, *P=0.021*; Table 2). As all subjects with a −670 AA genotype had a −1377 GG genotype, the −1377G/−670A ancestral haplotype was associated with a higher CD95 expression level than the −1377A/−670G haplotype. These data suggest that a genetic predisposition could contribute to CD95 overexpression in MDS patients.

### CD95 expression level at diagnosis is predictive of response to ESA, but not predictive of overall or event-free survival

We then investigated the impact of CD95 expression on clinical presentation at diagnosis, response to ESA treatment and survival in 162 lower risk MDS patients (Table 1). We have previously established that the threshold of CD95 expression predictive of MDS was a RFI of 1.7 in comparison with a large series of controls. [19] Here we found that 100/162 patients (61.7%) had a CD95 RFI ≥ 1.7, further denominated as CD95^high^ patients. There was no significant difference of age, sex ratio, past history of chemo or radiotherapy, blood parameters, bone marrow parameters, dysplasia, karyotype, IPSS and IPSS-R between CD95^high^ and CD95^low^ patients (Table 1). However, the representation of WHO subtypes was unbalanced (Fisher's exact test; *P* = 0.011), with a more frequent representation of RCMD in CD95^high^ patients than in CD95^low^ patients.

**Table 1:** Clinical and biological parameters of 162 low/int-1 MDS patients according to CD95 expression level

Parameters	CD95 cohort (n=162)	CD95^low^ cohort (n=62)	CD95^high^ cohort (n=100)	*P*
**Demography**				
Age (median [IQR25-75%])	73 [67-80]	74 [69 - 78]	72 [66 - 80]	0.854
Sex ratio M/F	1.6 (100/62)	1.5 (37/25)	1.7 (63/37)	0.672
Past history of chemo or radiotherapy yes/no (%)	16/123 (11.5)	9/42 (17.7)	7/81 (8.0)	0.084
**WHO 2008 % n (%)**				
5q- syndrome/RA	39 (24.1)	19 (30.7)	20 (20.0)	0.011*
RARS/RCMD-RS	44 (27.1)	21 (33.9)	23 (23.0)	
RCMD	33 (20.4)	5 (8.1)	26 (26.0)	
RAEB1	45 (27.9)	17 (27.3)	28 (28.0)	
MDS-U	1 (0.6)	0 (0)	1 (1)	
**Blood parameters (median [IQR])**				
Hemoglobin g/dL	9.9 [9.0 - 10.7]	9.9 [9.2 - 10.7]	10.0 [8.9 - 10.9]	0.985
Mean corpuscular volume fL	101 [93 - 107]	100 [90 - 108]	102 [94 - 107]	0.153
Neutrophils G/L	2.1 [1.2 - 3.6]	2.4 [1.4 - 3.7]	2.0 [1.2 - 3.1]	0.393
Platelets G/L	212 [126 - 296]	225 [108 - 323]	199 [133 - 274]	0.627
Reticulocytes G/L	47.3 [29 - 59.6]	47 [24 - 63]	49 [29 - 58]	0.921
**Bone marrow parameters**				
Richness n (%) poor/medium/high	11 (7.7) / 76 (53.2) / 56 (39.2)	5 (9.8) / 28 (54.9) / 18 (35.3)	6 (6.5) / 48 (52.2) / 38 (41.3)	0.661*
Blasts median (% [IQR])	3 [2 - 5]	3 [2 - 5]	3 [2 - 5]	0.736
Erythroblasts median (% [IQR])	29 [20 - 41]	28 [22 - 39]	31 [20 - 42]	0.783
**Bone marrow dysplasia yes/no (%)**				
Dyserythropoiesis	105/32 (76.6)	37/12 (75.5)	68/20 (77.3)	0.815
Dysgranulopoiesis	88/37 (70.4)	29/15 (65.9)	59/22 (72.8)	0.418
Dysmegakaryopoiesis	76/49 (60.8)	24/20 (54.6)	52/29 (64.2)	0.291
**Karyotype normal (yes/no)**	104/58 (64.2)	41/21 (66.1)	63/37 (63.0)	0.686
**Karyotype IPSS n (%)**				
Good	129 (83.8)	52 (86.7)	77 (81.9)	0.787*
Intermediate	24 (15.6)	8 (13.3)	16 (17.0)	
Poor	1 (0.6)	0 (0)	1 (1.1)	
**Karyotype IPSS-R n (%)**				
Very good	11 (6.9)	4 (6.5)	7 (7.2)	0.787*
Good	128 (80.5)	52 (83.9)	76 (78.4)	
Intermediate	18 (11.3)	6 (9.7)	12 (12.4)	
Poor	0 (0)	0 (0)	0 (0)	
Very poor	2 (1.3)	0 (0)	0 (2.1)	
****IPSS n (%)****				
low	93 (57.4)	40 (64.5)	53 (53.0)	0.151
int-1	69 (42.6)	22 (35.5)	47 (47.0)	
**IPSS-R n (%)**				
Very good (0-2)	45 (28.3)	21 (33.9)	24 (24.7)	0.396*
Good (>2-5)	87 (54.7)	33 (53.2)	54 (55.7)	
Intermediate (>5-7)	22 (13.8)	8 (12.9)	14 (14.4)	
Poor (>7-9)	4 (2.5)	0 (0)	4 (4.1)	
Very poor (>9-18)	1 (0.6)	0 (0)	1 (1.0)	
**Treatments yes/no (%)**				
RBC transfusions	74/63 (54.0)	25/27 (48.1)	49/36 (57.7)	0.275
Erythropoiesis-stimulating agents	118/23 (83.7)	43/9 (82.7)	75/14 (84.4)	0.807
Lenalidomide	35/92 (27.6)	13/36 (26.5)	22/56 (28.2)	0.837
Demethylating agents	25/108 (18.8)	7/44 (13.7)	18/64 (22.0)	0.238
Low dose aracytine	4/129 (3.0)	1/50 (2.0)	3/79 (3.7)	1.000
Intensive chemotherapy	3/126 (2.3)	0/49 (0)	3/77 (3.8)	0.288
Allo HSC transplantation	3/126 (2.3)	1/48 (2.0)	2/78 (2.5)	1.000
**Response to ESA (yes/no)**	75/43 (63.6)	33/10 (76.7)	41/34 (54.7)	0.017
**Overall survival in months(median [95%CI]) (n=156)**	108.1 [75.5 - 128.4]	111.1 [75.5 - ***]	90.2 [64.1 - 128.4]	

The threshold for CD95 RFI was 1.7. Continuous variables are expressed as median [interquartile range 25-75% or 95% confidence interval] and analyzed by using Student t-test or ANOVA test. Non parametric variables are expressed as percentages and analyzed by using Chi-square or Fisher's exact tests. *P* values <0.05 were considered as significant.

* for Fisher's exact test. IQR: interquartile range; CI: confidence interval; WHO: World Health Organization; RA: refractory anemia; MDS-U: undefined MDS; RARS: refractory anemia with ring sideroblasts; RCMD: refractory cytopenia with multilineage dysplasia; RCMD-RS: RCMD with ring sideroblasts; RAEB1: RA with excess of blast lower than 10%; RBC: red blood cells; HSC: hematopoietic stem cell; IPSS: International Prognosis Scoring System; IPSS-R: IPSS-revised.

**Table 2:** CD95 expression levels according to CD95 gene polymorphisms of the core promoter −1377 G>A and −670 A>G

*CD95* gene SNP	n (%)	CD95 RFI median [IQR 25-75]	*P*
−1377 GG	102 (79.1)	2.1 [1.6 - 2.7]	0.118
−1377 GA/AA	27 (20.9)	1.6 [1.6 - 2.5]	
−670 AA	32 (24.8)	2.5 [1.7 - 2.9]	0.021
−670 AG/GG	97 (75.2)	2.0 [1.4 - 2.6]	

Genotypes were determined for 129 MDS patients. Median CD95 RFI were compared using Mann-Whitney test. *P* values < 0.05 were considered as significant. IQR: Inter-Quartile Range.

**Figure 2:**
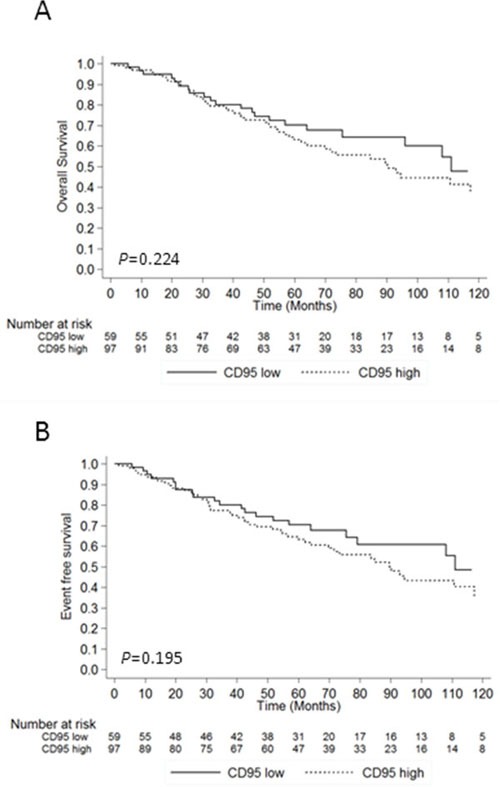
Impact of CD95 expression on survival and event-free survival **A.** Overall survival according to CD95 RFI in 153 MDS patients. **B.** Event-free survival according to CD95 RFI. Data are plotted as a Kaplan-Meier curve. Log Rank test for *P* values.

In the whole cohort, follow-up information was available for 204/250 (81.6%) and the median follow-up was 57.2 (IQR: 25.6 – 87.3) months. Clinical follow-up data were available for 156/162 (96.3%) low/int-1 MDS patients including 97 (62.2%) with high CD95 expression and 59 (37.8%) with low CD95 expression. The median follow-up in the low/int-1 cohort was 60.0 (IQR: 32.5 – 90.7) months. As shown in Table 1, treatments including RBC transfusions, ESA, lenalidomide, azacitidine, low dose aracytine, intensive chemotherapy and allo-HSCT were equivalent in CD95^low^ and CD95^high^ patients. No difference of overall survival (*P* = 0.224) or event-free survival (*P* = 0.195) was found between the two groups (Figure 2A & 2B). Finally, none of the studied genotypes at −1377 or −670 loci had any impact on overall survival or event-free survival (Supplementary Figure S2).

One hundred and eighteen patients were treated with ESA and 75/118 (63.6%) were responders after 3 months of treatment according to IWG2006 criteria. CD95^low^ patients (33/43; 76.7%) were more frequently responders to treatment than CD95^high^ patients (41/75; 54.7%; *P* = 0.017; Table 1). In univariate analysis, the predictors of ESA resistance were serum EPO level over 100 IU/mL (available for only 80 patients; *P* = 0.008) and previous transfusion (*P* = 0.001). To further explore the link between ESA response and C95 expression, we used a logistic regression to adjust on the presence or absence of previous transfusion. High CD95 expression level (OR: 2.5 [95%CI: 1.1 – 6.0], *P* = 0.036) and previous transfusion (OR: 2.7 [95%CI: 1.1 – 6.3], *P* = 0.025) remained independent predictors of ESA resistance.

### Effects of APG101 on erythroblast proliferation, differentiation and apoptosis

**Figure 3:**
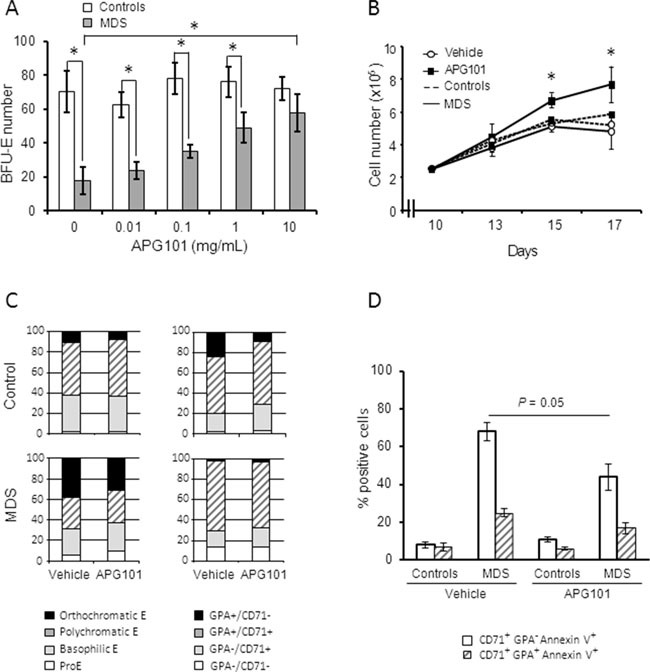
APG 101 improves the proliferation of erythroid progenitors of BFU-E type and of erythroid precursors by inhibiting apoptosis Erythroblasts were derived from CD34+ progenitors in liquid culture (controls, n=3; MDS, n=5 including 2 RA, 2 RCMD, 1 RARS). **A.** Cells were harvested at day 5 of the culture and seeded at 10^4^ cells/mL in the presence of increasing concentrations of APG101 in methylcellulose medium for colony assays. BFU-E were quantified after 10 days. Controls plotted as white bars, MDS as gray bars. **B.** APG101 (10 μg/mL) was added and renewed every two days from day 10 to 17. Amplification rate was evaluated by cell counting every two days. Controls as dotted lines, MDS as full lines. **C.** Erythroid cell differentiation was assessed at day 12 by cytological examination of May-Grünwald-Giemsa-stained cytospins (left panel) or by flow cytometry using a double labeling with CD71 and CD235a to glycophorin A (GPA) antibodies (right panel). Results are representative of 3 experiments. **D.** Apoptosis was quantified in CD71^+^ GPA^−^ and CD71^+^ GPA^+^ cell populations by flow cytometry in 5 cases of MDS and 3 controls. Results expressed as means ± SEM. Mann-Whitney test for *P* values. *: < 0.05.

We have shown here that CD95 overexpression was predictive of lower response rate to ESA in the present cohort of 118 low/int-1 MDS patients treated by ESA. Thus, an alternative therapeutic strategy must be used to cure anemia in these patients. In this aim, we investigated the effect of APG101, a soluble dimer of the extracellular domain of human CD95 in fusion with the Fc domain of human IgG1. Bone marrow erythroid progenitors from 5 patients with MDS (2 RA, 2 RCMD, 1 RARS) and 3 controls were expanded until day 9. [4] In all MDS cases, the expression of CD95 (RFI) was a range between 1.8 and 2.7. CD95 expression was higher in MDS [range: 1.7 – 2.8] compared to controls [range: 0.8 – 1.0] and increased during MDS erythroid progenitor amplification while it remained lower in control cultures (not shown). At day 5 of the liquid culture, erythroid progenitors of BFU-E-type were quantified in clonogenic assays in the presence of increasing concentrations of APG101. As shown in Figure 3A, mean BFU-E number was significantly lower in MDS compared to controls. APG101 induced a dose-dependent increase of BFU-E growth in MDS samples but not in the controls. BFU-E number reached the number of BFU-E in normal controls at a APG101 concentration of 10 μg/mL. From day 10 until day 17, we added APG101 (10 μg/mL) to the liquid culture to investigate the impact of the drug on erythroblast differentiation driven by erythropoietin and insulin. We observed that APG101 improved the proliferation rate without a significant effect on the kinetic of differentiation (Figures 3B & 3C). The repartition of the different erythroid precursor categories identified either by cytological examination of MGG-stained cytospins or flow cytometry was similar in the presence of APG101 or the control vehicle (Figure 3C). By contrast, APG101 reduced by 30% the level of apoptosis of immature precursors while it did not demonstrate any effect on the apoptotic level of mature precursors (Figure 3D). These results show that APG101 inhibited erythroblast apoptosis thus rescuing erythroid cell growth.

### APG101 effects on hematopoietic progenitor growth *in vitro*

Considering the rescue of BFU-E harvested from liquid culture by APG101, we further evaluated the effects of increasing concentrations of APG101 on BMMNC-derived clonogenic progenitors in 20 MDS patients (3 5q- syndrome, 3 RA, 1 RARS, 2 RCMD-RS, 5 RCMD, 6 RAEB1) and 5 controls. Among these patients, 15 received ESA and 14 were primary (n=6) or secondary (n=8) resistant to this treatment. The mean baseline number of BFU-E was significantly different (5.4 ± 1.3 colonies *per* 10^5^ BMMNC) in 15 MDS further referred as “low” compared to controls (33.5 ± 12.9 colonies *per* 10^5^ BMMNC; Mann-Whitney, *P* = 0.005) while in 5 cases (3 RCMD/RCMD-RS, 2 RAEB1) further referred as “normal”, the mean number of BFU-E (38.2 ± 5.5 colonies *per* 10^5^ BMMNC) was similar to that of controls (Mann-Whitney, *P* = 0.429). In these 15 cases (11 5q- syndrome/RA/RCMD, 4 RAEB1), CFU-E and CFU-GM numbers at baseline were also significantly lower than that of controls and “normal” MDS (Supplementary Figure S3) while CFU-L number was as low as in the controls group and lower than in the “normal” MDS group. There was no significant difference of WHO classification or CD95 expression between the 15 patients with low erythropoiesis at baseline *versus* the 5 patients with normal initial erythropoiesis (Fisher's exact test; *P=0.612)*. Eleven among 15 patients with low erythropoiesis (73.3%) received ESA and all of them were resistant to this treatment.

**Figure 4:**
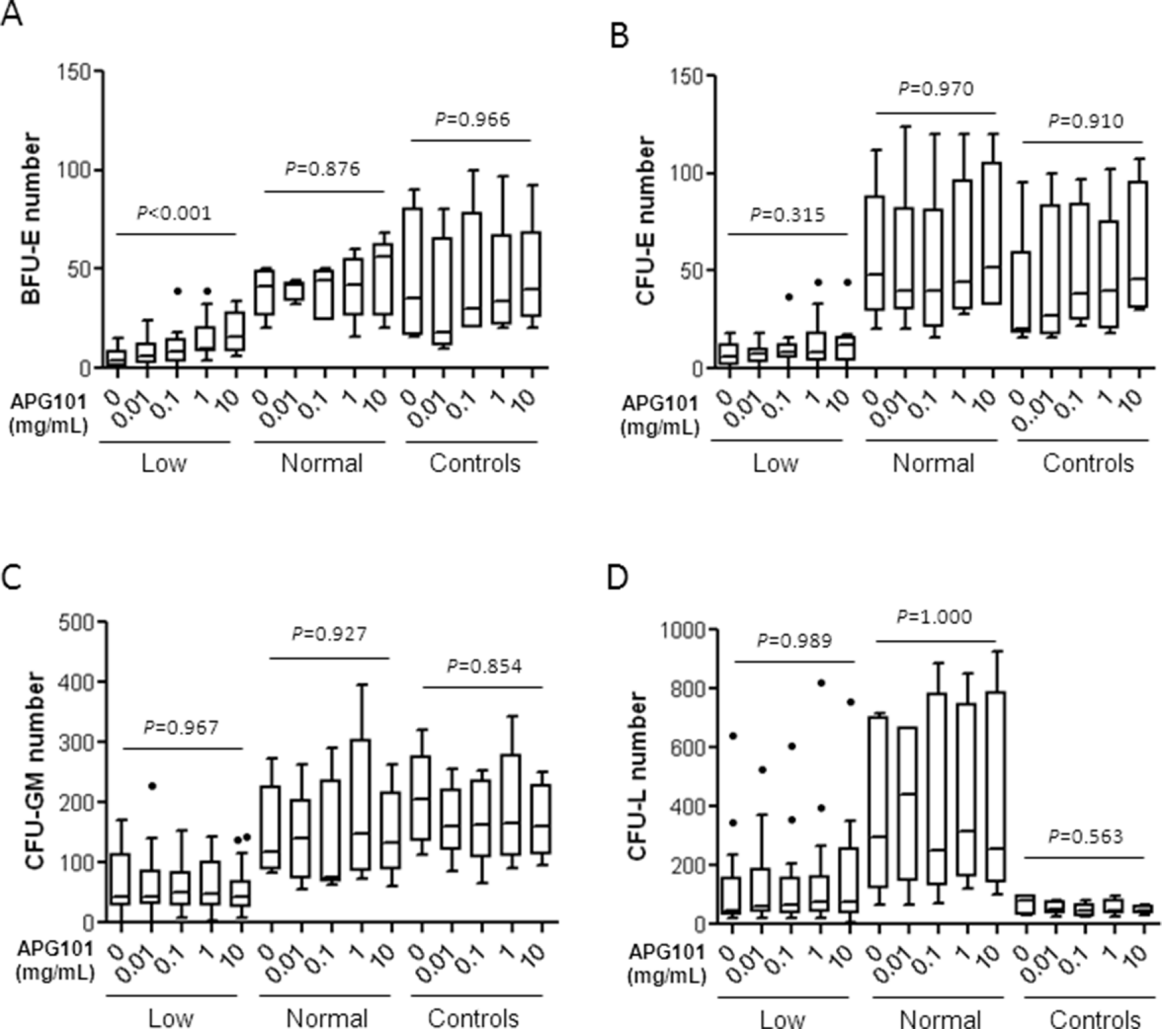
Effects of APG101 on the growth of hematopoietic progenitors Bone marrow mononuclear cells (3 5q- syndrome, 3 RA, 1 RARS, 2 RCMD-RS, 5 RCMD, 6 RAEB1, 5 controls). were seeded at 10^5^ cells/mL in methylcellulose medium in the presence of increasing concentrations of APG101. BFU-E and CFU-GM were counted at day 14 and CFU-E and CFU-L were counted at day 7. “Low” MDS (n=15) were defined as MDS with a significant lower BFU-E number compared to controls, and “normal” MDS (n=5) were defined as MDS with a BFU-E number equivalent to controls. Results are expressed as colony numbers. Horizontal bars represent medians and 1^st^ and 99^th^ centiles. Kruskal-Wallis test for *P* values.

APG101 induced a dose-dependent increase of BFU-E in these “low” MDS, but neither in the “normal” MDS nor in the controls group (Kruskal-Wallis test; *P*<0.001; Figure 4A). The number of CFU-E, CFU-GM, and more interestingly the number of CFU-L corresponding to leukemic cell clusters remained unchanged in every subgroup. (Figure 4B – 4D). The presence of a low number of BFU-E at baseline was significantly associated with *in vitro* response to APG101 among the 20 MDS patients (Fisher's exact test, *P* = 0.031) and also among the 14 ESA-resistant patients (*P* = 0.027).

### Low clonogenic progenitor number at baseline, but not CD95 or CD95L expression level is predictive of the response to APG101

**Figure 5:**
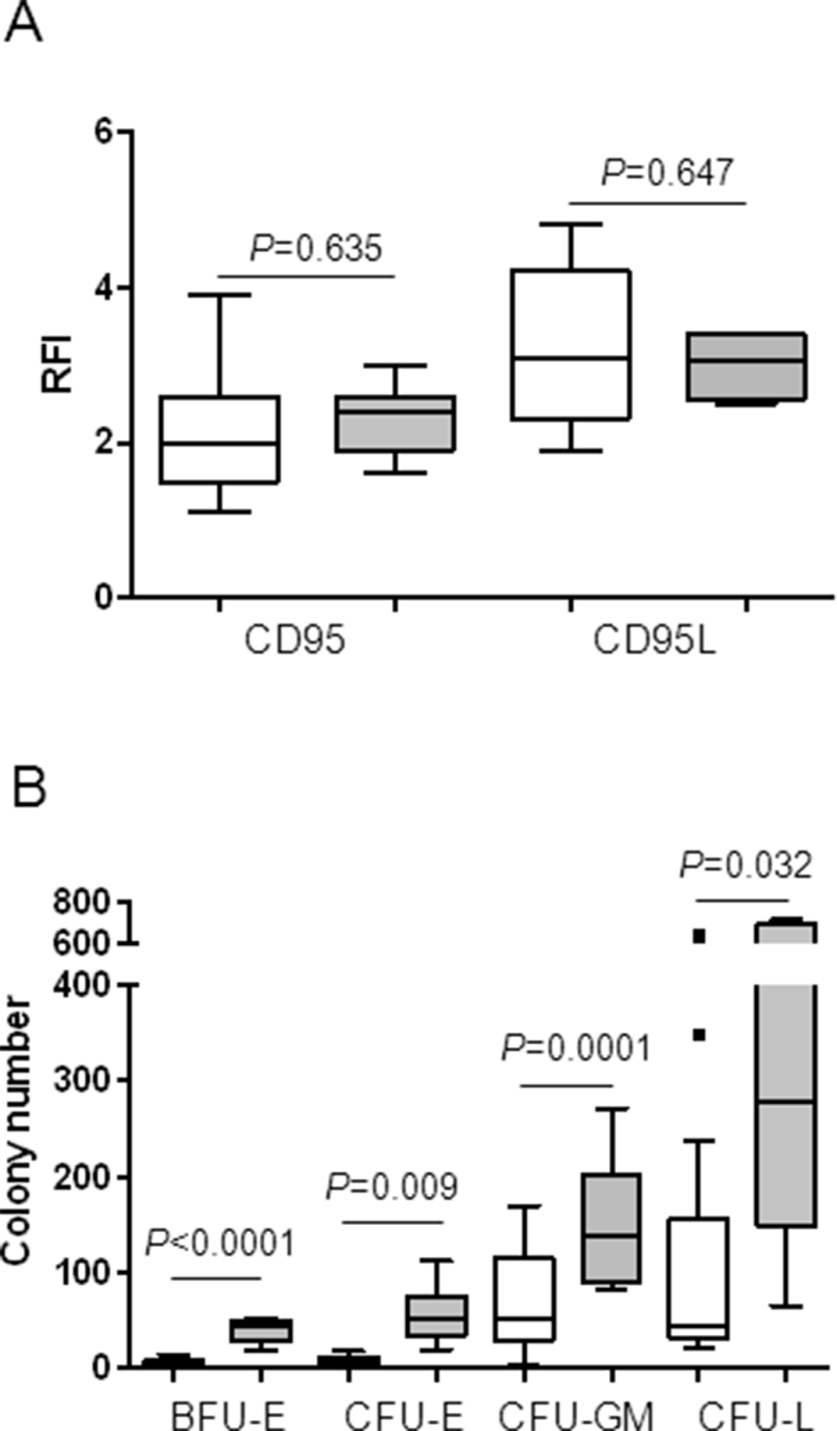
Low progenitor cell number, but not CD95 or CD95L expression at baseline is predictive of the response to APG101 **A.** CD95 and CD95L were quantified by flow cytometry and expressed as RFI. **B.** Bone marrow mononuclear cells were seeded at 10^5^ cells/mL in methylcellulose. BFU-E and CFU-GM were counted after 14 days and CFU-E and CFU-L were counted after 7 days. Results are expressed as colony numbers. White boxes for responders and gray boxes for non-responders. Horizontal bars represent medians and 1^st^ and 99^th^ centiles. Student t-test for *P* values.

Then, we compared CD95 and CD95L expression in the two groups of responder and non-responder patients to *in vitro* treatment with APG101. As shown in Figure 5A, the median CD95 RFI and the median CD95L RFI were not significantly different between MDS samples in which APG101 improved BFU-E growth compared to those in which it did not. By contrast, baseline number of BFU-E, CFU-E, CFU-GM and CFU-L was significantly lower in responders, suggesting that APG101 could rescue erythropoiesis in patients which hematopoiesis is severely impaired. Thus, we identified low clonogenic progenitor number at baseline as a good predictor of the response to APG101 (Figure 5B).

## DISCUSSION

In the present work, we show that CD95 overexpression is a biomarker of ESA resistance. Furthermore, patients with ESA resistance exhibit a severe impairment of their hematopoiesis. [13] APG101, a CD95 fusion protein, efficiently rescues the growth of MDS immature erythroid progenitors of BFU-E type when their baseline number is low, independently of the expression level of CD95 or CD95L.

Transcription of CD95 expression is genetically determined. The presence of an A nucleotide at −1377 or a G nucleotide at −670 disrupts the docking of transcription factors SP1 or STAT1 to *CD95* promoter, respectively. [16] Therefore, a lower transcription efficacy is observed when −1377A and −670G alleles are expressed. Here, we showed the genetic predisposition of patients with −1377G/−670A haplotype to a higher CD95 expression level than patients with −1377A/−670G haplotype. The genetic polymorphism −1377G>A in the core promoter of *CD95* gene that reduces the expression level of CD95 has been associated with the risk of developing AML. [16] This polymorphism also predicts a worse prognosis in acute promyelocytic leukemia. [20] CD95 expression is epigenetically regulated. We and others have reported that CD95 expression decreases due to hypermethylation and chromatin enrichment of repressive marks at the *CD95* promoter core as MDS disease progresses to AML. [19, 21] These two mechanisms of regulation are not mutually exclusive and may support the difference of CD95 expression between early MDS and secondary AML. However, CD95 expression does not affect event-free survival suggesting that variation of CD95 level would not be a unique determinant for MDS disease progression to AML.

We show that expression of membrane-bound CD95L on CD45^low^ cells was equivalent in MDS compared to controls. Previous works reported that soluble CD95L was not increased in MDS peripheral blood. [22, 23] Furthermore, the percentage of CD95L positive cells could be lower in MDS peripheral blood compared to healthy controls. [24] In contrast, increased membrane CD95L expression on myeloid blasts, erythroblasts and maturing myeloid cells of MDS bone marrow has been reported. [25] The reason for this discrepancy with our results showing a normal level of CD95L on immature CD45^low^ cells could be due to technical issues. Indeed, immunohistochemical staining of bone marrow samples used in the Gupta's study could be less quantitative than flow cytometry. CD95 is overexpressed on immature erythroblasts but also on CD34^+^ progenitors including leukemic blasts. [4] Therefore, trapping CD95 ligand using APG101 could protect blast cells from apoptosis. We report here that CD95L is not overexpressed on CD45^low^ and CD71^+^ myeloid precursors (not shown) in MDS marrow, while it is upregulated on maturing erythroblasts. [4] Of note, the granulo-monocytic lineage would more predominantly express the TNF-related apoptosis-inducing ligand (TRAIL) and its receptors. [5, 35] Furthermore, *in vitro* treatment with APG101 does not provoke a raise of CFU-L and CFU-GM colonies. The overall selectivity of APG101 for CD95L is higher by at least a factor of 100 than for other apoptosis inducing ligands of the tumor necrosis factor superfamily (LIGHT, TRAIL and TNFα). Therefore, our results suggest that leukemic progenitors and myeloid precursors other than erythroblasts are not target cells for APG101.

Alternative therapeutic strategies are needed to treat anemia in ESA-resistant lower risk MDS patients. Lenalidomide has been shown to induce ESA response in 30% of cases with an improvement of the response rate up to 50% when erythropoietin (EPO) was combined to lenalidomide. [26] But, half of the patients remained non-responders to this second line of treatment. Interestingly, the activin IIB ligand trap, luspatercept, gave promising results in a phase 2 study designed to correct ineffective erythropoiesis in low/int-1 ESA-resistant patients. [27] In the present preclinical study, we propose APG101 in the treatment of anemia in patients with severe impairment of erythropoiesis. APG101 rescued BFU-E growth in MDS samples and increased the number of differentiated precursors by reducing apoptosis. Caspases are massively activated when MDS erythroblasts enter the final phase of maturation, [28] while normal erythroid cell differentiation requires a limited activation of caspases. [29, 30] We have previously demonstrated that controlling the level of caspase activation by expressing a dominant negative Fas-associated death domain (FADD) or Bcl-2 does not impair the differentiation capacities of MDS erythroblasts. [5, 31] Here, we show that APG101 preserves the capacity of MDS erythroblasts to differentiate *in vitro* while reducing their apoptotic level. In other pathological situations, activation of CD95 pathway protects cells from death. CD95 or CD95L invalidation may induce a necrotic death closed to a mitotic catastrophe in ovarian or liver cancer cells. [32] Furthermore, CD95 stimulates the metastatic dissemination in progressive glioblastoma and a phase II clinical trial shows that invasion can be blocked by weekly administration of APG101. [33, 34] The interim report of a phase I clinical trial of APG101 in 5 low/int-1 transfusion-dependent MDS patients receiving weekly intravenous infusions of 100 mg or 400 mg APG101 for 12 weeks reported an increase of BFU-E/CFU-E and CFU-GM progenitors, no increase of medullary blasts after a 6-month follow-up, no side effects, and a decrease of transfusion frequencies in 4 patients. [36] These encouraging data must be confirmed on a larger cohort of patients.

In conclusion, we have shown that overexpression of CD95 at diagnosis is a hallmark of ESA resistance and that severe impairment of hematopoiesis is predictive of erythroid response to APG101 *in vitro*. Altogether, these data provide a rationale for further clinical investigation.

## MATERIALS AND METHODS

### Patients

MDS patients (n=250) attending Cochin hospital were prospectively enrolled between January 2004 to July 2012 in a monocentric observational study, after they gave informed consent according to the local ethics committee recommendations. Inclusion criteria were a diagnosis of MDS according to the WHO classification and a sample collection at diagnosis and before disease-modifying treatments. Patients with idiopathic cytopenia of undetermined significance or with AML were excluded. WHO classification was 5q- syndrome/refractory anemia (RA)/MDS-U n=54; RA with ring sideroblasts (RARS)/refractory cytopenia with multilineage dysplasia (RCMD) and ring sideroblasts n=56; RCMD n=51; RA with excess of blasts type 1 (RAEB1) n=66, and RAEB type 2 (RAEB2) n=23. IPSS was available for 192 patients and 162 (84.4%) patients were classified as lower risk MDS, *i.e*. low/int-1 IPSS (Supplementary Table S1). Follow-up information of including the date of death or AML transformation, defined as a bone marrow blast count ≥ 20%, was obtained at each follow-up consultation until the 24^th^ November 2014. Patient status was recorded in the national MDS registry (http://test.registre-smd.clinfile.com). One hundred and thirty-nine MDS patients received ESA and the response was evaluated in 118 low/int-1 patients according to the International Working Group criteria. [14] CD95 expression was studied in the whole cohort while CD95L was studied in a subgroup of 82 patients. DNA samples from a subgroup of 129 patients were available for the study of CD95 gene promoter polymorphisms. Control samples (n=30) were from patients matched in age suffering of anemia of other origin than MDS, either inflammation or cancer invasion. Patients with thalassemia or aplastic anemia were excluded.

### Flow cytometry

CD95 expression was quantified by direct labeling using the fluorescein isothiocyanate (FITC)-coupled CD95 antibody (clone UB-2, Beckman Coulter, Fullerton, CA). CD95 ligand (CD95L) was quantified using the biotinylated mouse anti-human CD95L (CD178) antibody (clone NOK-1, Pharmingen, Becton Dickinson) followed by streptavidin-coupled to phycoerythrin (PE). Results were expressed as ratio of median fluorescence intensities (RFI) between specific and isotype-matched antibodies within gated CD45^low^ bone marrow cells. Analyses were performed using a Cytomics FC500 apparatus (Beckman Coulter) with CXP software.

### CD95 gene promoter polymorphisms

Bone marrow mononuclear cells (BMMNC) were isolated on Ficoll gradient. DNA was extracted using commercial kits (Qiagen, Hilden, Germany). Polymorphisms located at −1377nt (rs2234767) and −670nt (rs1800682) of the 5′-UTR of the CD95 gene was analyzed by PCR and Sanger sequencing on ABI 3300 capillary sequencer (Agencourt Bioscience, Beverly, MA, USA) using the primers: CD95_RS1377_F: 5′-CCCTCCTT CCATTCCTTCTTCCC-3′ and CD95_RS1377_R: 5′-C CCTTTGCT TAGCCCACTGTTC-3′, CD95_RS670_F: 5′-AGCTGGGGCTATGCGATTTG-3′ and CD95_RS 670_R: 5′-GAGCCTTGGCTAATTGCTGG-3′. Mutation Surveyor was used for analysis (SoftGenetic©, Inc., Stat College, PA, USA).

### Colony assays

Colony assays were performed in 20 low/int-1 MDS patients, including 15 patients treated by ESA, 14 of them being primary or secondary resistant to this treatment and 5 controls were isolated on Ficoll gradient and seeded in methylcellulose containing IL3, SCF, EPO, GM-CSF and G-CSF at 10^5^ cells/mL (Stemcell technologies, Vancouver, Canada) with 0 to 10 μg/mL APG101. Colony assays were validated by an annual external quality control. Progenitors were counted at day 7 for colony-forming unit-erythroid (CFU-E) and colony-forming unit-leukemic (CFU-L) and at day 14 for burst-forming unit-erythroid (BFU-E) and colony-forming unit-granulomonocytic (CFU-GM).

### Liquid culture

CD34^+^ progenitors (MDS = 5; controls = 3) were purified from BMMNC using the MidiMac system (Miltenyi Biotech, Bergisch Gladbach, Germany), and expanded to the erythroid lineage in the presence of erythropoietin (EPO), stem cell factor (SCF), insulin-like growth factor-1 (IGF-1) and dexamethasone until day 10.^4^ At day 5 of the liquid culture, cells were harvested and seeded in methylcellulose with 0 to 10 μg/mL APG101. BFU-E and CFU-GM were counted after 10 days. From day 10 of the liquid culture, erythroid cell maturation was obtained by replacing SCF, IGF-1 and dexamethasone by insulin. APG101 (10 μg/mL) was added and renewed every two days. Cells were counted and erythroid cell differentiation was assessed on May-Grünwald-Giemsa-stained cytospins and flow cytometry using CD71-FITC and glycophorin A (GPA)-PE antibodies. Apoptosis was quantified using annexin V-allophycocyanin (APC) (Becton-Dickinson).

### Statistical analysis

For continuous variables, values were expressed as median and interquartile range (IQR) or 1-99 centiles and were compared using Student t, Mann-Whitney, ANOVA or Kruskal-Wallis tests. Categorical variables are reported as counts and percentages and compared using Chi-squared or Fisher's exact tests. Multivariate analysis regarding the response to ESA was performed using logistic regression and results were expressed as odds ratio and 95% confidence intervals (95% CI). Survival time was computed from the time of MDS diagnosis to the time of the death for overall survival (OS) or to the time of AML transformation for event free survival (EFS). Censoring occurred in individuals who were alive (and without AML transformation for EFS) at the 24^th^ November 2014, or who were lost from follow-up before that time. Survival was evaluated by the Kaplan-Meier method and comparisons were carried out with the Log-Rank test. Statistical tests were bilateral and a *P*-value<0.05 was considered to indicate statistical significance. Analyses were performed using the GraphPad Prism v.6 (GraphPad Software, La Jolla, CA) and Stata version 12 (StataCorp L.P., College Station, TX).

## SUPPLEMENTARY FIGURES AND TABLE


