# Supplementary material for: APG101 efficiently rescues erythropoiesis in lower risk myelodysplastic syndromes with severe impairment of hematopoiesis
Source: Oncotarget. 2016 Feb 18;7(12):14898–911. doi: 10.18632/oncotarget.7469 (PMC4924760; doi:10.18632/oncotarget.7469)
Supplement: Supplementary file 1 [file oncotarget-07-14898-s001.pdf]

SUPPLEMENTARY FIGURES AND TABLE

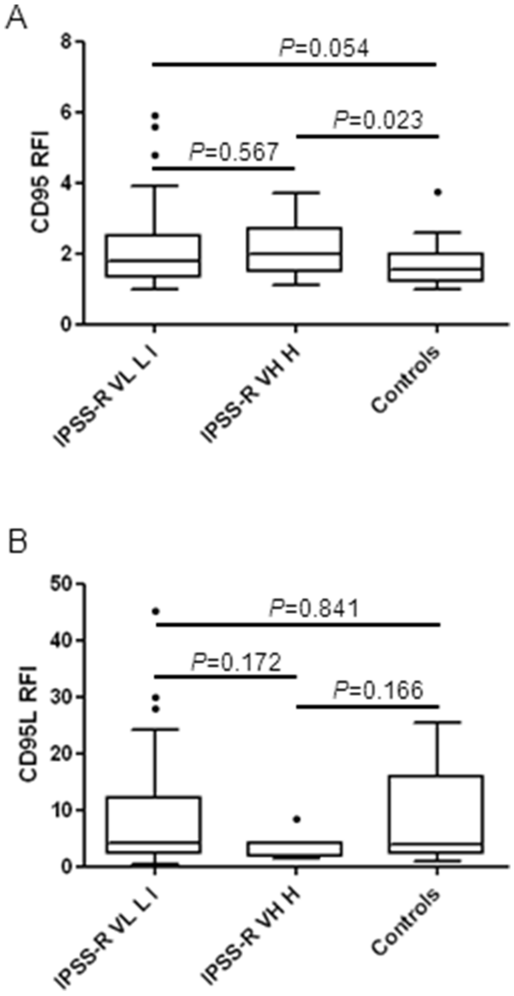

Supplementary Figure S1: CD95 and CD95L expression level according to IPSS-R. Quantification by flow cytometry in 250 MDS patients and 30 controls for CD95 expression and in 82 MDS patients and 18 controls. Medians were compared between IPSS-R very low/low/intermediate (VL L I), IPSS-R very high/high (VH H) and control groups using Student *t*-test. *P* values < 0.05 were considered as significant.

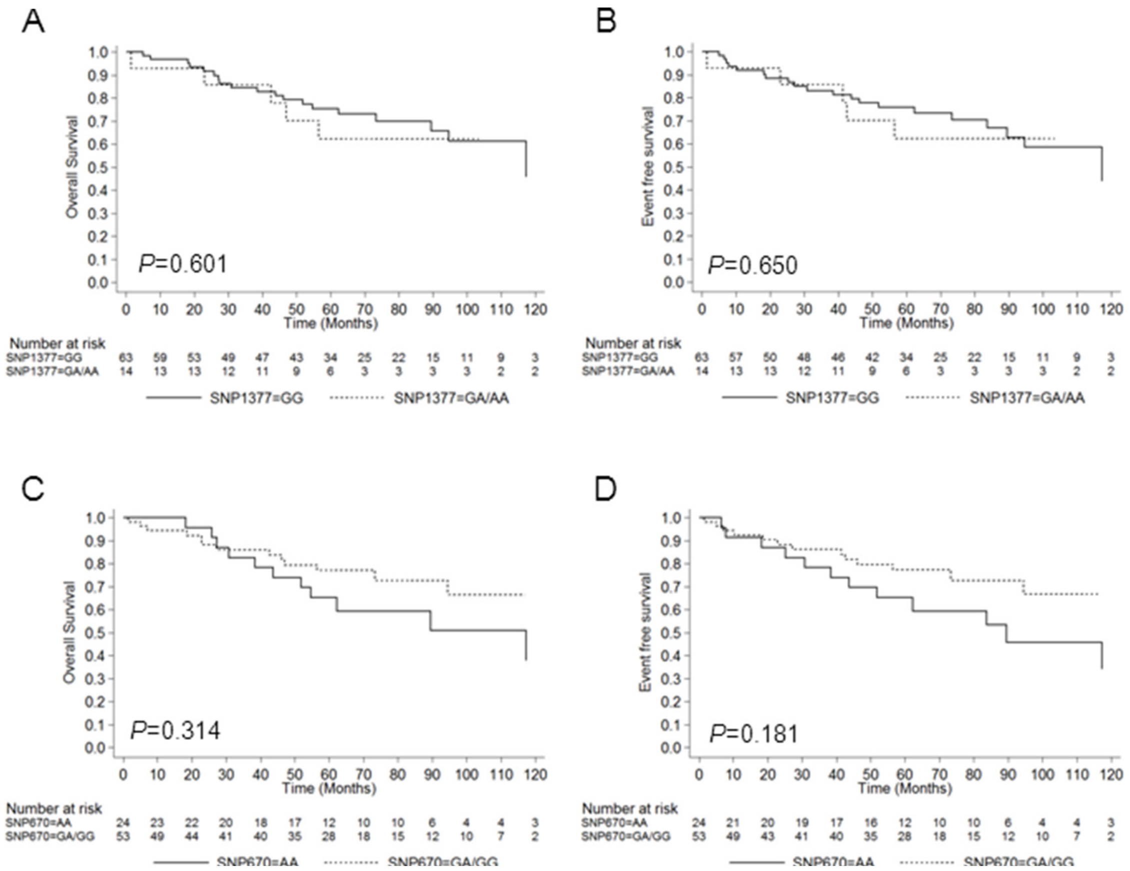

Supplementary Figure S2: Overall survival and event-free survival in 105 MDS patients according to -1377 G>A **A. & B.** or -670 A>G **C. & D.** polymorphisms. Data are plotted as a Kaplan-Meier curves. Log Rank test for P values.

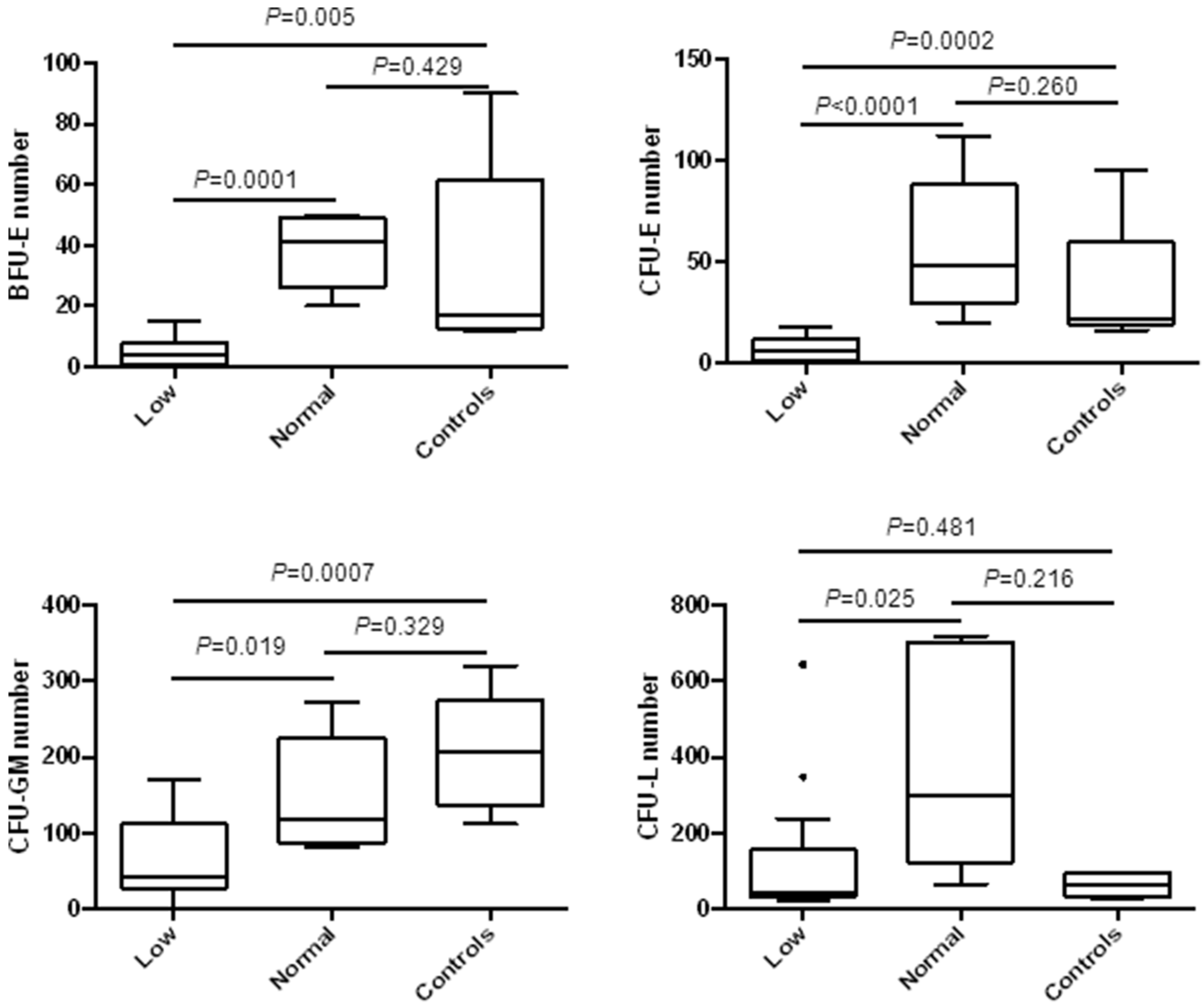

Supplementary Figure S3: Hematopoietic progenitors at baseline. BFU-E, CFU-E, CFU-GM and CFU-L were quantified in methylcellulose assays and expressed as colony numbers. Results from 6 controls and 20 low/int-1 MDS including 15 "low" MDS characterized by a significantly lower colony number than controls and 5 "normal" MDS which colony numbers equivalent to that of controls. Horizontal bars represent medians and 1st and 99th centiles. Kruskal-Wallis test for *P* values.

Supplementary Table S1: Clinical and biological parameters of MDS patients

Parameters	CD95 cohort (*n* = 250)	CD95L cohort (*n* = 82)
**Demography**		
Age at diagnosis median [IQR25–75%]	73 [66 - 80]	74 [64 - 79]
Sex ratio M/F	1.6 (155/95)	1.5 (50/32)
Past history of chemo or radiotherapy yes/no (%)	30/150 (16.7)	10/43 (18.9)
**WHO 2008 n (%)**		
5q- syndrome/RA/MDS-U	54 (21.6)	12 (14.7)
RARS/RCMD-RS	56 (22.4)	23 (28.0)
RCMD	51 (20.4)	23 (28.0)
RAEB1	66 (26.4)	19 (23.2)
RAEB2	23 (9.2)	5 (6.1)
**Blood parameters median [IQR]**		
Hb g/dL	9.9 [8.9 - 10.7]	10.1 [9.0 - 11.2]
MCV fL	100 [92 - 107]	101 [96 - 107]
Neutrophils G/L	2.0 [1.2 - 3.2]	3.5 [2.9 - 5.7]
Platelets G/L	184 [103 - 275]	163 [92 - 267]
Reticulocytes G/L	47 [29 - 61]	45 [21 - 57]
**Bone marrow parameters**		
Richness n (%) poor	18 (9.4)	4 (6.8)
medium	102 (53.1)	34 (57.6)
high	72 (37.5)	21 (35.6)
Blasts median % [IQR]	3 [2 - 6]	3 [2 - 5]
Erythroblasts median % [IQR]	29 [20 - 40]	28 [18 - 43]
Dyserythropoiesis n (%)	129/50 (72.1)	40/9 (81.6)
Dysgranulopoiesis n (%)	115/52 (68.8)	35/13 (72.9)
Dysmegakaryopoiesis n (%)	103/64 (61.7)	27/21 (56.3)
**Karyotype normal yes/no (%)**	116/80 (59.2)	28/23 (54.9)
**Karyotype IPSS n (%)**		
Good	142 (75.5)	38 (74.5)
Intermediate	30 (16.0)	7 (13.7)
Poor	16 (8.5)	6 (11.8)
**Karyotype IPSS-R n (%)**		
Very good	12 (6.2)	4 (8.2)
Good	141 (73.1)	33 (67.4)
Intermediate	23 (11.9)	5 (10.2)
Poor	5 (2.6)	3 (6.12)
Very poor	12 (6.2)	4 (8.2)

(*Continued*)

Parameters	CD95 cohort (*n* = 250)	CD95L cohort (*n* = 82)
**IPSS n (%)**		
low	93 (48.5)	27 (54.0)
int-1	69 (35.9)	14 (28.0)
int-2	26 (13.5)	8 (16.0)
high	4 (2.1)	1 (2.0)
**IPSS-R n (%)**		
Very high (0-2)	45 (23.8)	14 (28.6)
High (>2-5)	88 (46.6)	21 (42.9)
Intermediate (>5-7)	29 (15.3)	7 (14.3)
Low (>7-9)	13 (6.9)	4 (8.2)
Very low (>9-18)	14 (7.4)	3 (6.1)
**Treatments yes/no (%)**		
RBC transfusions	98/75 (56.6)	23/21 (52.3)
Erythropoiesis-stimulating agents	139/44 (76.0)	36/15 (70.6)
Lenalidomide	39/120 (24.5)	12/34 (26.1)
Demethylating agents	43/126 (25.4)	16/30 (34.8)
Low dose aracytine	6/164 (3.5)	1/45 (2.2)
Intensive chemotherapy	9/153 (5.5)	0/46 (0.0)
allo HSC transplantation	4/158 (2.5)	1/45 (2.2)
**Response to ESA yes/no (%)**	86/50 (63.2)	20/12 (62.5)

CD95 RFI was quantified in a cohort of 250 MDS patients and CD95L in a cohort of 82 patients. Continuous variables are expressed as median [interquartile range 25–75%]. IQR: interquartile range; WHO: World Health Organization; RA: refractory anemia; MDS-U: undefined MDS; RARS: refractory anemia with ring sideroblasts; RCMD: refractory cytopenia with multilineage dysplasia; RCMD-RS: RCMD with ring sideroblasts; RAEB1: RA with excess of blast lower than 10%; RAEB2: RAEB ≥ 10%; IPSS: International Prognosis Scoring System; IPSS-R: IPSS-revised; RBC: red blood cells; HSC: hematopoietic stem cell.
